# #infertility education on Instagram: A Reliability Analysis in Brazil Using the DISCERN Instrument

**DOI:** 10.5935/1518-0557.20260006

**Published:** 2026

**Authors:** Márcia Mendonça Carneiro, Renata Bossi, Debora Alvarenga, Ana Carolina Xavier, Rodrigo Hurtado, Marcos Sampaio, Marisa Mendonça Carneiro

**Affiliations:** 1 Department of Gynecology and Obstetrics of the School of Medicine of the Federal University of Minas Gerais, Belo Horizonte, MG, Brazil; 2 ORIGEN Reproductive Medicine Center, Belo Horizonte, MG, Brazil; 3 Faculty of Letters of the Federal University of Minas Gerais, Belo Horizonte, MG, Brazil

**Keywords:** social media, infertility, patient education

## Abstract

**Objective:**

To assess the prevalence, authorship, and reliability of educational fertility-related information shared on Brazilian Instagram, measured by the DISCERN instrument.

**Methods:**

This cross-sectional study analyzed public fertility-related educational Instagram posts published in March 2021 related to the following hashtags: #infertility, #ivf, #endometriosis, #tryingtoconceive, #maternity, #humanreproduction, #pregnancy, #invitrofertilization, #assistedreproduction, #pregnant, #difficulttogetpregnant. Educational posts were evaluated using the DISCERN tool. Authorship was categorized as either healthcare professional (HCP) or layperson (LP).

**Results:**

The majority of the 37 analyzed posts were authored by HCP (n=33; 89.2%), with 24 of these being physicians, five fertility clinics, four allied HCP, one magazine, and three LP. Most posts (n=15) focused on fertility treatments; other topics included information about diseases, exams, and diagnosis. The mean Discern analysis scores for each question were: (1) Are the aims clear? 4.86; (2) Does it achieve its aims? 4.75; (3) Is it relevant? 4.47; (4) Is it clear what sources of information were used to compile the publication (other than the author or producer)? 1.58; (5) Is it clear when the information used or reported in the publication was produced? 1.59; (6) Is it balanced and unbiased? 1.59; (7) Does it provide details of additional sources of support and information? 1.04; (8) Does it refer to areas of uncertainty? 1.39.

**Conclusions:**

Although physicians authored most posts that clearly stated the aim and relevance, important issues such as the source of information used and details about additional sources of support and information were not available. The posts analyzed here failed to be balanced and unbiased and did not inform about potential areas of uncertainty.

## INTRODUCTION

Infertility rates have been rising, and according to the World Health Organization (WHO), it affects one in every six people worldwide (WHO, 2023), while global fertility rates are declining, raising concerns about the consequences of underpopulation (Fauser *et al.*, 2025). Since access to diagnosis and treatment is available to only a few fortunate individuals (Njagi *et al.*, 2023; Norman & Fauser, 2024), many people on the infertility journey turn to online resources for information (Broughton *et al.*, 2018). In fact, social media has become a major source of health-related information for many seeking fertility care, and the numbers have increased in recent years. People search social media for health-related information for various reasons, which may include: learning about a specific disease or physician/hospital, obtaining guidance or a second opinion, and even sharing experiences and connecting with others facing the same condition (Farsi *et al.*, 2022).

Studies have shown that individuals facing infertility often turn to social media platforms like Instagram and Facebook to seek information, find fertility clinics, share experiences, and even get second opinions (Sormunen *et al*., 2020; Peyser *et al*., 2021). The COVID-19 pandemic may have enhanced the role of social media as a source of information for fertility-related issues (Perone *et al*., 2020). Unfortunately, in the fertility space on social media, physicians and academic societies are the minority and less influential than patients, support groups, and the so-called influencers, who comprise the majority according to Blakemore *et al.* (2020).

Brazilians are particularly keen on using social media for health information. Sadly, the quality and accuracy of the daily content posted are controversial and need to be assessed. Recent published research shows social media is increasingly used not only for information and education but also for emotional and social support, as well as searching for fertility clinics and doctors (Perone *et al.*, 2021; Carneiro *et al.*, 2024). Since fertility care information is limited and infertility rates are rising, social media may serve as an alternative for those seeking care and information. Therefore, we aimed to determine the prevalence, authorship, and reliability of educational fertility-related information shared on Instagram in Brazil using the DISCERN instrument according to (Logullo *et al.*, 2019).

## MATERIAL AND METHODS

This cross-sectional study examined publicly available posts related to infertility hashtags on Brazilian Instagram, the most popular social media platform among reproductive-aged people in Brazil (Bianchi, 2025).

A list of 11 hashtags related to fertility terms commonly used on Instagram was selected based on (TagsFinder.com, 2025): #infertility, #ivf, #endometriosis, #tryingotconceive, #maternity, #humanreproduction, #pregnancy, #invitrofertilization, #assistedreproduction, #pregnant, #difficulttogetpregnant. To minimize the influence of the platform’s algorithm, a new Instagram account (@resmedgob) was created. Authorship was categorized as either healthcare professionals (HCP) or laypeople (LP). Posts were collected in March 2021, with the 20 most recent ones included in the analysis. Only educational posts written in Portuguese were evaluated, while video and commercial posts were excluded. Educational posts included those providing education or information on fertility topics such as infertility diagnosis or workup, endometriosis, and other conditions. Two authors used the DISCERN (Logullo *et al.*, 2019) tool to assess the quality and reliability of the posts, and any disagreements were reconciled. The DISCERN instrument consists of 16 questions divided into three sections: reliability (1-8), quality of information on treatment choices (9-15), and overall rating (16). Each question was scored from 1 to 5, with 1 indicating no agreement and 5 indicating the highest agreement. A score of five was assigned when the quality criterion was fully met, while scores of 2, 3, or 4 indicated partial fulfillment of the criterion. A score of 1 was used when the criterion was not met at all.

For this study, questions 1 to 8 were used to assess the reliability of the shared information: (1) Are the aims clear? (2) Does it achieve its aims? (3) Is it relevant? (4) Is it clear what sources of information were used to compile the publication (other than the author or producer)? (5) Is it clear when the information used or reported in the publication was produced? (6) Is it balanced and unbiased? (7) Does it provide details of additional sources of support and information? (8) Does it refer to areas of uncertainty?

Three specific questions focus on key aspects for the evaluation: question 4 addresses the ability to verify the information; question 6 is used to identify bias in the post, and question 7 pertains to the connection with additional sources. DISCERN reliability scores can be interpreted as follows: 1-2.9 = poor reliability; 3-4 = moderate reliability; and >4 = high reliability.

This study does not require approval from an Ethics Committee because it used publicly available information and did not involve any interaction or intervention with human subjects, in accordance with local regulations (Ministério da Saúde - Conselho Nacional de Saúde, 2016).

## RESULTS

Thirty-seven posts met the inclusion criteria and were analyzed. Most were authored by healthcare professionals (n=33; 89.2%). The authors included 24 medical doctors, five fertility clinics, four allied healthcare professionals, one magazine, and three laypeople. The majority of posts (n=15) focused on fertility treatments; other topics covered diseases such as endometriosis, exams, and diagnoses. Mean Discern analysis for each question is shown in Table 1.

**Table 1:** Mean Discern Analysis.

Discern question	Mean score
(1) Are the aims clear?	4.86
(2) Does it achieve its aims?	4.75
(3) Is it relevant?	4.47
(4) Is it clear what sources of information were used to compile the publication (other than the author or producer)?	1.58
(5) Is it clear when the information used or reported in the publication was produced?	1.59
(6) Is it balanced and unbiased?	1.59
(7) Does it provide details of additional sources of support and information?	1.04
(8) Does it refer to areas of uncertainty?	1.39

## DISCUSSION

Our results show that the majority of the posts were authored by healthcare professionals, with medical doctors responsible for the majority of educational infertility-related content on Instagram. Unfortunately, the reliability of these posts, as measured by the DISCERN instrument, was poor when questions 4 to 8 were considered. Although the posts clearly stated their aim and relevance, they lacked important details such as the sources of information used, additional sources of support, and other relevant details. The analyzed posts also failed to be balanced and unbiased, and most importantly, they did not address areas of uncertainty even though they were primarily authored by physicians.

People on the fertility journey often use social media to share their experiences and seek support, but healthcare professionals, including physicians, are increasingly using it as a low-cost educational platform (Peyser *et al.*, 2021). In Brazil, Instagram is a common source for health-related information and may provide a great opportunity to educate patients and their families about infertility (Carneiro *et al.*, 2024). I analyzed infertility posts on X and Instagram using various tools, including an adaptation of DISCERN, and found that these posts often lack accuracy, credibility, and reliability. Since social media platforms are important sources of information on a complex issue like fertility, there is a need to focus on the quality and trustworthiness of these posts (Perone *et al.*, 2021; Dhanoya *et al.*, 2025).

Since the information available on these platforms can influence how people make decisions about health and disease, ensuring this material is reliable is of utmost importance. Therefore, individuals should recognize that, given the vast amount of content posted by nearly anyone, much of it should be questioned since most posts are not verified (Carneiro, 2022). In fact, because so many posts are published daily, verifying accuracy is nearly impossible (Quaas, 2020; Carneiro, 2022). Additionally, people need to be aware of the risks posed by misinformation and commercial bias present in many so-called educational and scientific posts on social media, as well as the influence of so-called influencers who may have their own agenda (Quaas, 2020; Dhanoya *et al.*, 2025).

Unfortunately, there is a paucity of studies on the quality and reliability of infertility information shared on social media. Dhanoya *et al.* (2025) used Twitter/X and Instagram to examine the quality, credibility, accuracy, and readability of fertility-related posts. Most (74%) of these posts failed to cite scientific references, and unfortunately, 45% contained incorrect data while only 11% were considered reliable. Additionally, readability analysis showed that the majority (80%) were difficult to understand.

Instagram has approximately 122.9 million users in Brazil, with 42.39% males and 57.61% females (Bianchi, 2025). There is no denying the significant educational potential Instagram offers, as it can reach a large audience at low cost. Since more people are increasingly using social media as a source of health information and many may not have access to proper fertility care, they might base their decisions on the posts that are readily available, there is an urgent need to ensure these posts and profiles share clear, reliable content (Dhanoya *et al.*, 2025). Thus more studies are needed to understand and improve how Instagram is used in fertility/infertility care.

The DISCERN instrument was chosen because it is easy to use and has been validated in Portuguese (Logullo *et al.*, 2019). Unfortunately, there is a shortage of tools that have been properly validated to evaluate the quality of information available online (Alhlayl & Alzghaibi, 2025). It should be noted that the DISCERN tool cannot be used to assess the scientific quality or accuracy of the evidence on which a publication is based, as this would require searching and verifying other sources. However, it can assess the reliability of the shared information and evaluate whether the sources used are accurate. As social media use is rapidly increasing, the need for clear, accessible, and scientifically based information shared on social media cannot be overstated. The use of 11 common hashtags chosen according to (https://www.tagsfinder.com/) allowed for a comprehensive search on Instagram and the selection of relevant educational posts, providing a significant overview of fertility-related content shared on Brazilian Instagram. Since our aim was to evaluate the reliability of the shared information, we focused on questions 1 to 8.

To the best of our knowledge, this is the first time the accuracy and reliability of fertility-related Instagram posts have been analyzed in Brazil. Our analysis was limited to Portuguese and the number of posts included, but it may serve as a starting point for understanding the social media fertility landscape and improving the quality of information shared. More studies on the accuracy and reliability of infertility-related content posted on social media platforms like Instagram are needed.
